# Mechanisms of intermittent theta-burst stimulation attenuating nerve injury after ischemic reperfusion in rats through endoplasmic reticulum stress and ferroptosis

**DOI:** 10.1007/s11033-024-09241-x

**Published:** 2024-03-01

**Authors:** Xin-Ya Shen, Xing-Yu Zhang, Ping-Ping Han, Yi-Ning Zhao, Guo-Hui Xu, Xia Bi

**Affiliations:** 1 Department of Rehabilitation Medicine, Shanghai University of Medicine and Health Sciences Affiliated Zhoupu Hospital, Shanghai, China; 2 Graduate School of Shanghai, University of Traditional Chinese Medicine, Shanghai, China; 3 Department of Sport Rehabilitation, Shanghai University of Sport, Shanghai, China; 4 Huadong Hospital, Affiliated to Fudan University, 221 West Yan’an Road, Jing’an District, 200040, Shanghai, China

**Keywords:** iTBS, ERS, Ferroptosis, Neurological injury, Ischemic stroke

## Abstract

**Background:**

Repetitive transcranial magnetic stimulation (rTMS) exerts neuroprotective effects early in cerebral ischemia/reperfusion (I/R) injury. Intermittent theta-brust stimulation (iTBS), a more time-efficient modality of rTMS, improves the efficiency without at least decreasing the efficacy of the therapy. iTBS elevates cortical excitability, and in recent years it has become increasingly common to apply iTBS to patients in the early post-IS period. However, little is known about the neuroprotective mechanisms of iTBS. Endoplasmic reticulum stress (ERS), and ferroptosis have been shown to be involved in the development of I/R injury. We aimed to investigate the potential regulatory mechanisms by which iTBS attenuates neurological injury after I/R in rats.

**Methods:**

Rats were randomly divided into three groups: sham-operated group, MCAO/R group, and MCAO/R + iTBS group, and were stimulated with iTBS 36 h after undergoing middle cerebral artery occlusion (MCAO) or sham-operated. The expression of ERS, ferroptosis, and apoptosis-related markers was subsequently detected by western blot assays. We also investigated the mechanism by which iTBS attenuates nerve injury after ischemic reperfusion in rats by using the modified Neurological Severity Score (mNSS) and the balance beam test to measure nerve function.

**Results:**

iTBS performed early in I/R injury attenuated the levels of ERS, ferroptosis, and apoptosis, and improved neurological function, including mNSS and balance beam experiments. It is suggested that this mode of stimulation reduces the cost per treatment by several times without compromising the efficacy of the treatment and could be a practical and less costly intervention.

## Introduction

As a major cause of disability and death worldwide, stroke poses a great threat to human life. Depending on the site of damage, patients may experience a variety of sequelae, such as impaired motor, sensory, speech, and cognitive functions. Strokes are classified as ischemic and hemorrhagic, with ischemic being the majority [1]. Ischemic-hypoxic lesion necrosis occurs when cerebral blood vessels are obstructed and blood flow is significantly reduced resulting in inadequate oxygen supply [2]. The infarcted lesion consists of a central zone and a peripheral zone, which is the ischemic penumbra. Due to the presence of collateral circulation, the ischemic penumbra still receives part of the blood supply and the damaged nerve cells are in a reversible state [3]. Therefore, thrombolysis within 3 to 4.5 h after stroke can save the penumbra and effectively reverse tissue damage, and it is currently the main means of clinical treatment for ischemic stroke [4]. However, when ischemia and hypoxia are prolonged, the intracellular ion concentration balance is disrupted, calcium overload in neurons leads to the activation of multiple cell death pathways, and damaged neurons will not be salvaged. In addition, the restoration of blood supply may cause cerebral ischemia-reperfusion injury, which is associated with a variety of pathophysiological processes such as increased oxygen free radicals, intracellular calcium overload, and over-activation of inflammatory responses [5]. The narrow time window for thrombolysis and reperfusion injury results in poor functional recovery in patients with ischemic stroke; therefore, there is an urgent need to explore therapeutic strategies for IS to improve the symptoms and prognosis of the patients, which not only improves the quality of life of the patients but also reduces the economic burden on the society [6].

Due to the abundance of easily peroxidized cholesterol and unsaturated fatty acids in neurons and glial cell membranes, coupled with low levels of antioxidant enzymes in the brain, the ability to scavenge free radicals is low [7]. As a result, brain tissue is very susceptible to oxygen radical attack. The current study suggests that both endoplasmic reticulum stress (ERS) triggered by reactive oxygen species (ROS) and ferroptosis are involved in neurological damage after ischemic stroke. The endoplasmic reticulum (ER) is a central organelle involved in metabolic processes and is primarily responsible for protein synthesis and processing as well as maintaining intracellular calcium homeostasis. Aggregation of unfolded or misfolded proteins or an imbalance in intracellular calcium homeostasis can trigger ERS, a self-protective mechanism.ERS maintains protein homeostasis by triggering the unfolded protein response (UPR), which activates three stress sensors located in the endoplasmic reticulum membrane: serine/threonine-protein kinase/endoribonuclease inositol-requiring enzyme 1 α (IRE1), the protein kinase R -like endoplasmic reticulum kinase (PERK) and transcription factor 6 (ATF6) [8]. Moderate ERS facilitates the restoration of intracellular homeostasis; however, when the cell fails to restore homeostasis, excessive ERS triggers cell death by activating autophagy and apoptosis [9]. Ferroptosis is the latest cell death pathway discovered in 2012, and iron overload, lipid peroxidation, and glutathione peroxidase 4 (GPX4) inactivation are the three key elements that induce ferroptosis [10]. Brain tissue contains large amounts of iron, which plays an important role in the growth and development of the nervous system and is associated with synaptic development, myelin sheath formation, neurotransmitter Synthesis, and oxidative metabolism of neuronal cells [11], and extremely small disruptions in iron homeostasis can significantly alter the function of the organ. Excess free iron (Fe^2+^) catalyzes lipid peroxidation after cerebral ischemia, along with reduced glutathione (GSH) levels [12], which promotes the onset of ferroptosis, leading to neuronal death [13].

Transcranial magnetic stimulation (TMS) is a painless and noninvasive magnetic stimulation technique that can produce clinical improvement in ischemic stroke [14, 15]. Repetitive Transcranial Magnetic Stimulation (rTMS) is a more commonly used clinical treatment [16]. As the name “rTMS” describes, it refers to the generation of more than two regular repetitive stimulation pulses at a time, and the magnetic signals can reach the cerebral cortex through the skin and skull without attenuation, causing changes in the membrane potential of cortical nerve cells [17]. Low-frequency rTMS (LF-rTMS) with a frequency ≤ 1 Hz inhibits cortical function, while high-frequency rTMS (HF-rTMS) with a frequency > 1 Hz excites cortical function [18]. Recently, it has been shown that rTMS burst stimulation mode (TBS) has similar or even better efficacy than conventional rTMS [19]. It combines multiple pulses into a single string stimulation, which largely shortens the treatment time compared to rTMS [19]. Two different stimulation modalities, intermittent theta-burst stimulation (iTBS) and continuous theta-burst stimulation (cTBS) produce opposite effects. iTBS increases cortical excitability, whereas cTBS decreases cortical excitability [20]. However, it is not clear whether TBS can alleviate nerve damage by inhibiting ERS and ferroptosis. In the present study, we investigated the MCAO/R model in rats treated with iTBS ipsilateral to the lesion to assess the neuroprotective effects of iTBS by attenuating ERS and ferroptosis and to explore the potential value of iTBS application in reperfusion injury in ischemic stroke.

## Methods

### Experimental animals

All animal experiments were in accordance with the National Institutes of Health Guide for the Care and Use of Laboratory Animals (promulgated by the National Research Council in 1996), and the protocols were approved by the Nursing Committee of the Institute of Laboratory Animal Research, Shanghai University of Traditional Chinese Medicine. Adult male Sprague-Dawley rats (weight 230–270 g, 6–8 weeks old) were purchased from Shanghai SLRC Laboratory Animal Co. The rats were housed under experimental conditions experiencing a 12-hour light and 12-hour dark cycle at a temperature of 22 ± 2 degrees Celsius, and were able to move freely with access to adequate food and water. After 7 days of acclimatization, the rats were randomly divided into three groups: the sham-operated group, the MCAO group, and the MCAO + iTBS group. The experimenter who performed the final evaluation was unaware of any allocation in the mouse experiments.

### Middle cerebral artery occlusion/reperfusion (MCAO/R)

Fasting was performed for 1 day before MCAO surgery and body weight was recorded before anesthesia. During surgery, rats were continuously anesthetized by 1.5% isoflurane, and body temperature was maintained by thermostatic pads. The rats were immobilized in the supine position, and the skin was incised by making a vertical incision along the middle of the clavicle after the neck was partially shaved. The left common carotid artery (CCA), internal carotid artery (ICA), and external carotid artery (ECA) were bluntly separated with a cotton swab until the left common carotid artery (CCA), internal carotid artery (ICA), and external carotid artery (ECA) were fully exposed. A ligature was made distal to the left ECA and a slipknot was tied proximally. The CCA and ICA were clamped with small arterial clips. The left ECA was straightened, and a suitable nylon ligature (Ruihua Life Science and Technology Co., Ltd., China) was slowly inserted into the stump of the ECA and then passed through the CCA into the ICA. When the ligature was inserted for 18–20 mm and resistance was felt, it indicated that the ligature had reached the middle cerebral artery, and the purpose of occlusion of blood flow could be achieved. At this time, the reserved ligature wire was tightened to provide a fixation effect, and 30 ml of saline was injected intraperitoneally for rehydration after suturing layer by layer. After 90 min of middle cerebral artery occlusion, the nylon thread plug was pulled out for reperfusion. No nylon wire pins were inserted in the sham operation group, and all other operations were consistent with those in the MCAO group. After awakening from anesthesia, Longa scoring was performed, and rats with a score of 1–3 were included in the experiment. Animals with a score of 4 showed severe brain damage and were excluded from the experiment.

### iTBS

The rats were randomized into three groups: the sham operation group, the MCAO/R group, and the MCAO/R + iTBS group. The operator was unaware of the grouping of the experiments. Rats in the MCAO/R + iTBS group were given a treatment regimen of iTBS 36 h after MCAO/R surgery [21]. The rats were stimulated twice a day for 7 consecutive days. Stimulation protocol: intensity 25%, intra-cluster frequency 50 Hz, intra-cluster count 3, intra-cluster stimulation time 0.06 s, inter-cluster frequency 5 Hz, inter-from count 10, stimulation time 2.00 s, intermittent time 8 s, repetitions 20 times, total number of stimuli 600. The sham-operated and MCAO/R groups were given sham stimulation, i.e., the coil was placed 15 cm above the head of the rat, which was beyond the effective stimulation range.

### Nissl and hematoxylin-eosin (HE) staining

Intact brain tissues were removed after rats were sacrificed, and then immersed in 4% paraformaldehyde and treated at 4 °C for 48 h. Paraffin sections with a thickness of 4 µM were made, which were subsequently deparaffinized, hydrated, rinsed, and stained with cresyl violet, and then dehydrated with 95% ethanol for 5 min, 100% ethanol for 10 min, and xylene for 10 min, and the changes were observed with a light microscope. Alternatively, tissues were sectioned and then deparaffinized with xylene, rehydrated with gradient ethanol, and stained sequentially with HE to observe pathological changes.

### Western blot assays

Infarcted tissue from rat brain cortex was taken and washed repeatedly, lysis buffer and protease inhibitor were added, and the upper layer of the clear liquid was centrifuged to make the protein sample to be tested. Protein concentration was determined by the BCA method, and according to the results obtained, the sample concentration was adjusted to be consistent and boiled for 10 min to denature the protein sufficiently. Subsequently, the proteins were separated by SDS-PAGE, and the proteins on the gel were transferred to polyvinylidene difluoride (PVDF) membranes for immunoblotting. The PVDF membranes were closed at room temperature for 2 h to improve specificity and sensitivity. Finally, the membranes were incubated in antibody dilutions overnight at 4 °C. IRE1 (1:500 dilution, Abclonal, A17940), Phospho-IRE1 (1:500 dilution, Abclonal, AP0878), PERK (1:1000 dilution, Cell signaling, 5683 S). Phospho-PERK (1:1000 dilution, Cell signaling, 3179 S), ATF6 (1:1000 dilution, Cell signaling, 65,880 S), GRP78 (1:1000 dilution, Abcam, ab21685), COX2 (1:1000 dilution, Cell signaling, 12,282 S), GPX4 (1:1000 dilution, Cell signaling, 59,735 S), CHOP (1:1000 dilution, Cell signaling, 2895 S), Bcl-2 (1:5000, Proteintech, 60178-1-Ig). Unbound primary antibody was washed three times with TBST buffer for 10 min each. The protein bands were then incubated with horseradish peroxide-labeled secondary antibody for 1 h at room temperature, washed three times similarly, and detected with chemiluminescent reagents. Image observation using an EPSON imaging system (EPSON; V300, Japan). Densitometric analysis was performed using Alpha software (Alpha Innotech; alphaEaseFC, USA).

### Neurobehavioral assessment

Conducted 24 h after MCAO/R surgery and after 7 consecutive days of iTBS stimulation, the severity of neurological deficits in rats was determined by scoring all rats using the modified Neurological Severity Score (mNSS). mNSS range from 0 to 18 and are divided into motor, sensory, and reflex components for testing. Among them, the motor includes muscle status and abnormal movement, and sensory includes vision, touch, and proprioception [22]. The higher the score, the more severe the neurological deficit. Rats with mNSS greater than 6 points for MCAO/R injury were included in the experiment.

### Evaluation of neurological function

Motor coordination and balance of rats were evaluated by the balance beam test, performed after 7 days of continuous stimulation with iTBS. The environment was ensured to be quiet and free of disturbance during training and testing. Wooden boards with a width of 2 cm and a length of 120 cm were fixed at each end to a support frame [23], so that they were kept horizontal and suspended 10 cm above the ground, and soft pads were provided under the balance beam to prevent the rats from falling and getting injured. Rats were trained to cross the balance beam every day for 3 days prior to MCAO/R and were encouraged to continue walking by tapping their rumps when they stalled. Rats that failed to cross the balance beam were not included in the experiment. Tests were performed 1 h after MCAO/R by placing the rat at one end of the balance beam. The number of falls and postural adjustments were observed and scored as the rats walked. The scoring was as follows [23, 24]: 0, no ability to stand and fall from the balance beam; 1, no movement but could stay on the balance beam; 2, attempted to walk but would fall and could not pass through the balance beam; 3, walked through the balance beam with more than 50% of the total number of steps with foot slips; 4, walked through the balance beam with less than 50% of the total number of steps with foot slips; 5, successfully passed through the balance beam with only one-foot slip on the affected side; 6, Successfully passed the balance beam without foot slip.

### Statistical analysis

IBM SPSS Statistics 25 statistical software and GraphPad Prism 9.0 were used in the study. data from the sham-operated group, MCAO/R group, and MCAO/R + iTBS group were analyzed by one-way analysis of variance (ANOVA), followed by the Tukey multiple comparison as a post hoc comparison, and Mann-Whitney U test for nonparametric analysis. Data were considered statistically significant when *P* < 0.05.

## Results

### iTBS attenuates ERS after cerebral ischemia-reperfusion

**Fig. 1:**
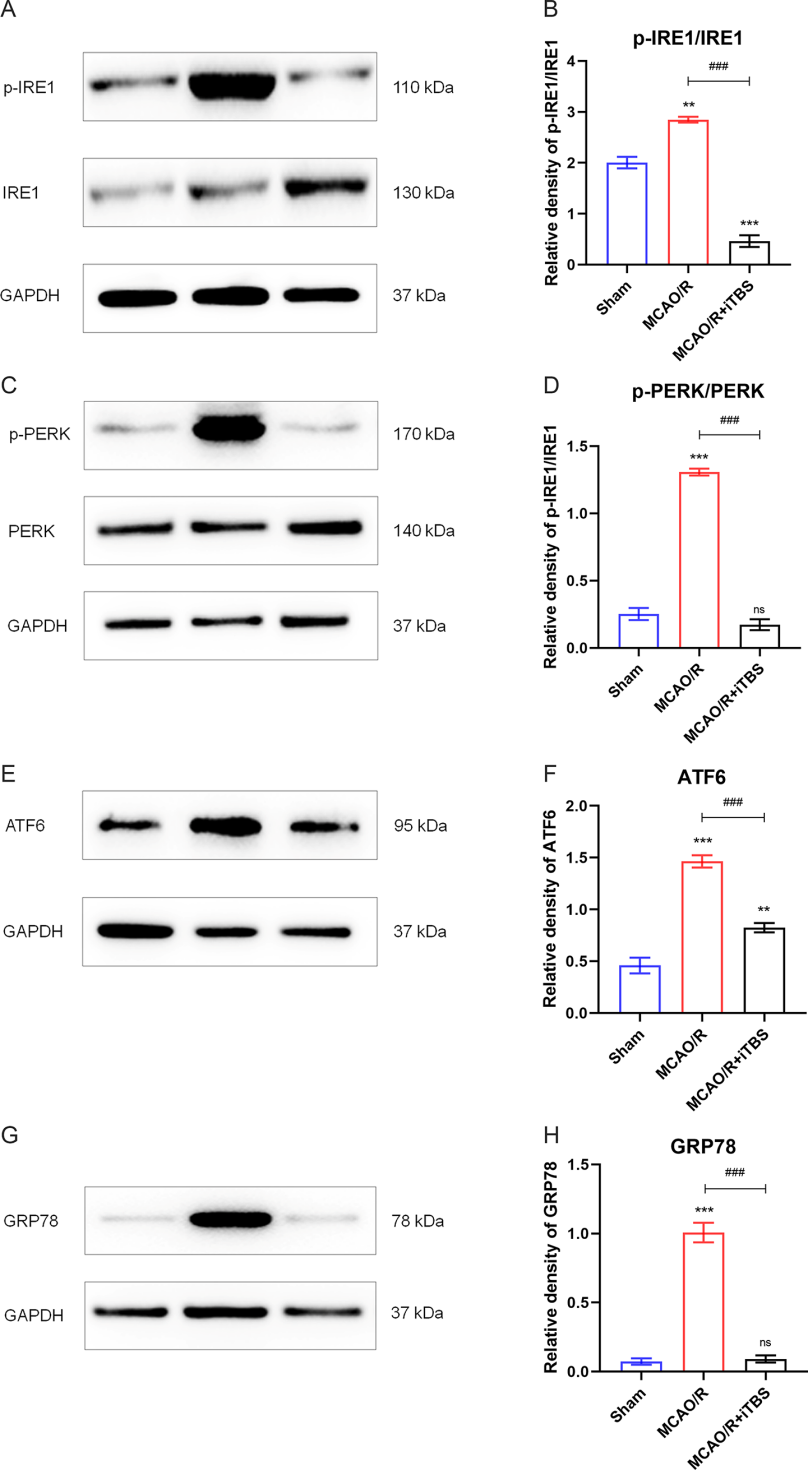
iTBS attenuated the occurrence of ERS after cerebral ischemia-reperfusion. (**A**-**H**) Representative protein blots of p-IRE1, IRE1, p-PERK, PERK, ATF6 and GRP78. Standardized to GAPDH. *n* = 6/group, ^**^P < 0.01 vs. Sham group, ^***^P < 0.001 vs. Sham group, ns indicates no statistical significance vs. Sham group, ^###^*P* < 0.001 vs. MCAO/R group. Data are expressed as mean ± standard deviation

We verified the effects of iTBS by examining the levels of IRE1, PERK, ATF6, and GRP78. Protein expression of IRE1, PERK, ATF6, and GRP78 was detected after MCAO/R as well as iTBS intervention. The results showed that MCAO/R rats had higher levels of IRE1, PERK, ATF6, and GRP78 compared to the sham-operated group, and the levels of IRE1, PERK, ATF6, and GRP78 were reduced in MCAO/R rats treated by iTBS (Fig. 1A-H). The above data suggest that neurologic deficits in MCAO/R rats are associated with ERS activation, and iTBS reversed ERS in MCAO/R rats.

### iTBS attenuates ferroptosis cerebral ischemia-reperfusion

**Fig. 2:**
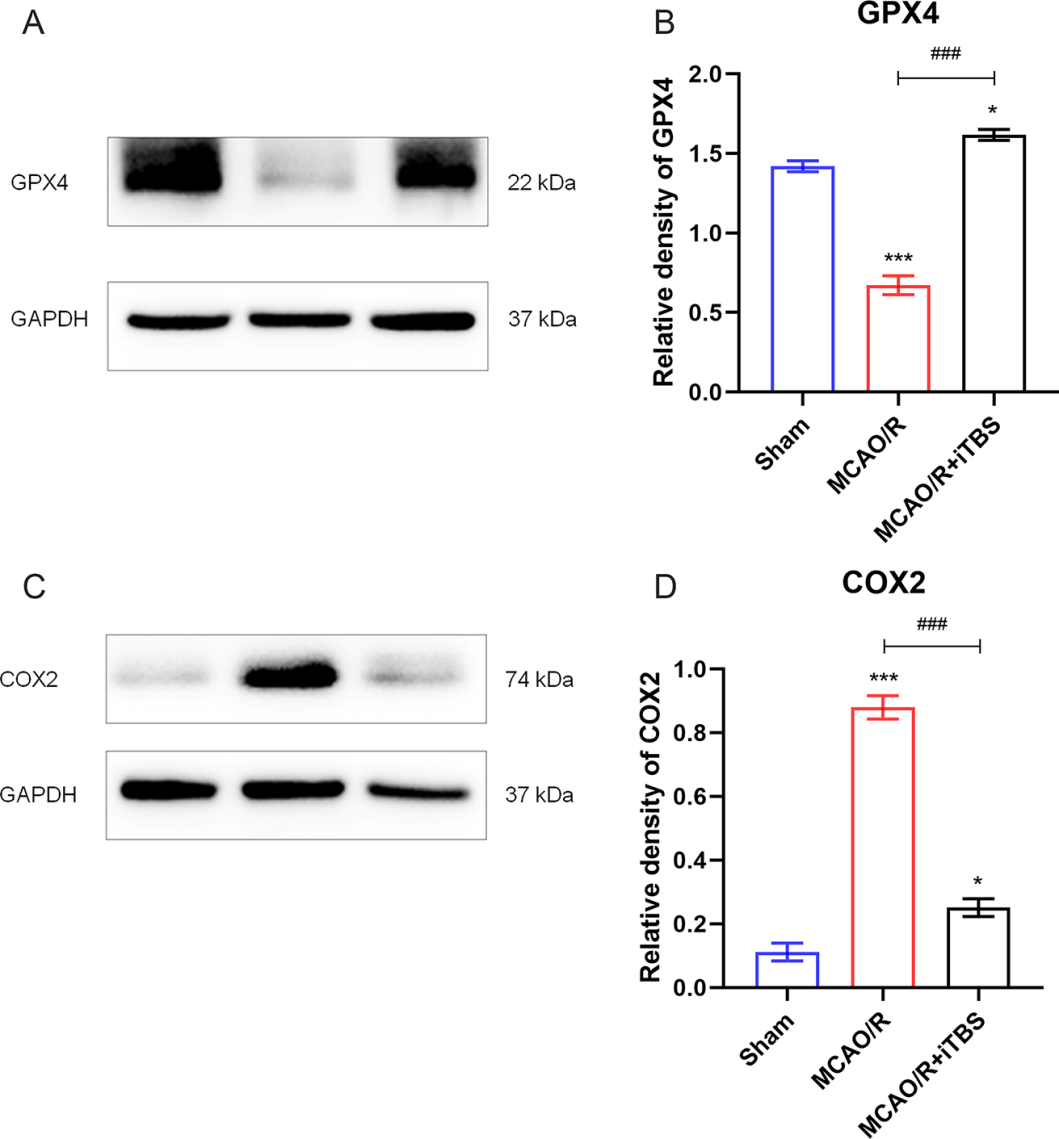
iTBS attenuated the occurrence of ferroptosis after cerebral ischemia-reperfusion. (**A**-**D**) Quantification of GPX4 and COX2 protein levels. GAPDH was used as an internal control. *n* = 6/group, ^*^P < 0.05 vs. Sham group, ^***^P < 0.001 vs. Sham group, ^####^*P* < 0.001 vs. MCAO/R group. Data are expressed as mean ± standard deviation

We explored whether iTBS treatment had an effect on ferroptosis. GPX4 and COX2 levels associated with ferroptosis were assessed by protein blotting. After cerebral ischemia, ferroptosis was significantly upregulated in rats in the MCAO/R group, as evidenced by decreased GPX4 levels and increased COX2 levels. However, the MCAO/R + iTBS group showed the opposite trend to the MCAO/R group, exhibiting increased GPX4 levels and decreased COX2 levels (Fig. 2A-D). The results indicated that iTBS treatment inhibited MCAO/R-induced ferroptosis.

### iTBS attenuates neuronal cell apoptosis in rats after cerebral ischemia-reperfusion

**Fig. 3:**
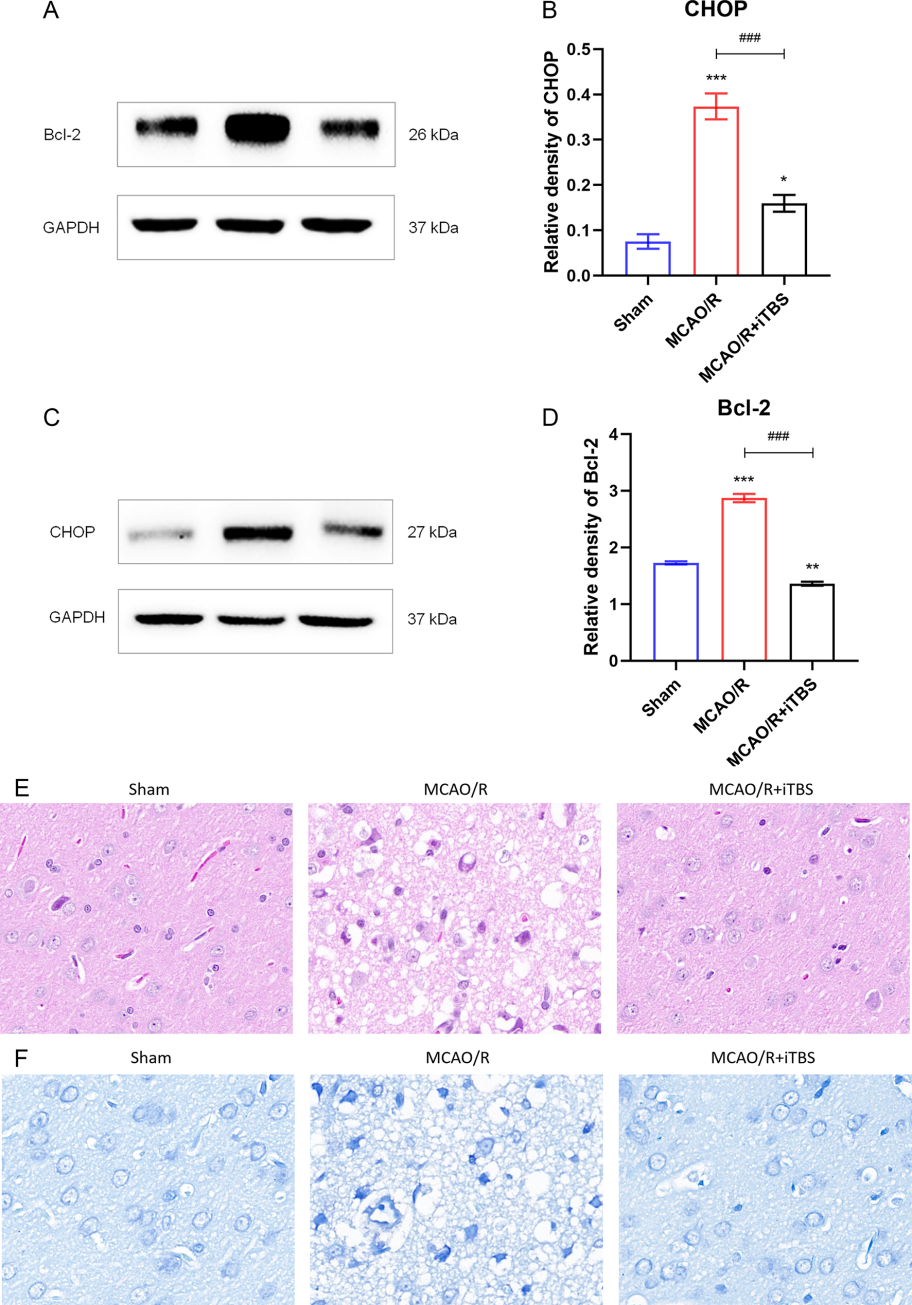
iTBS attenuates neuronal cell apoptosis in rats after cerebral ischemia-reperfusion. (**A**-**D**) Representative plots of CHOP and Bcl-2 protein bands. Standardized to GAPDH. *n* = 6/group, ^*^P < 0.05 vs. Sham group, ^**^P < 0.01 vs. Sham group, ^***^P < 0.001 vs. Sham group, ^###^*P* < 0.001 vs. MCAO/R group. Data are expressed as mean ± standard deviation. (**E**) Nissl staining shows the appearance of apoptosis. (**F**) HE staining shows histopathologic changes

After cerebral ischemic injury, the expression level of CHOP in rats of the MCAO/R group was increased, while the expression level of anti-apoptotic protein Bcl-2 was decreased. After treatment with iTBS, the level of CHOP was down-regulated, while the level of Bcl-2 was up-regulated (Fig. 3A-D). Consistently, Nissl staining showed increased neuronal cell apoptosis after MCAO/R and decreased neuronal cell apoptosis after iTBS treatment (Fig. 3E). In addition, HE staining also showed that the cells in the Sham group were neatly arranged with clear structures. cells in the MCAO/R group were irregularly arranged with blurred structures. Compared with the MCAO/R group, the pathological changes were significantly improved after iTBS treatment (Fig. 3F). It indicates that iTBS can alleviate apoptosis caused by continuous ERS after cerebral ischemia and has neuroprotective effects. The above data demonstrated that iTBS treatment could attenuate neuronal apoptosis in MCAO/R injury.

### iTBS attenuates neurological decline in rats after cerebral ischemia

**Fig. 4:**
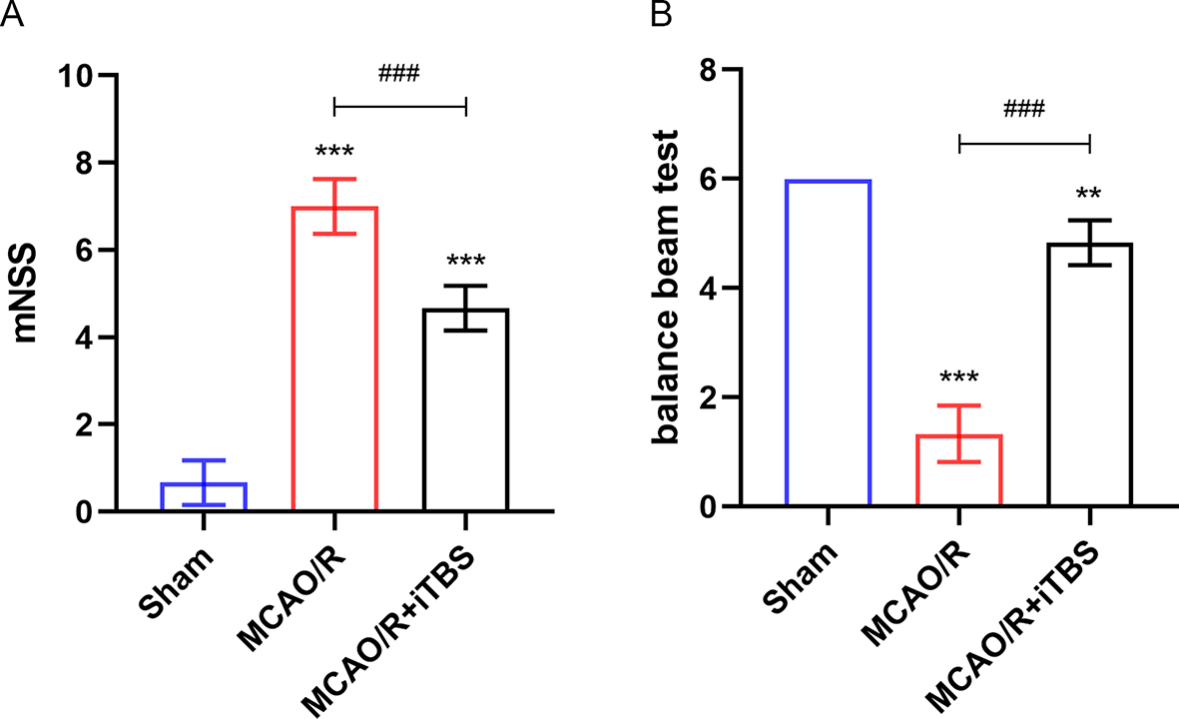
iTBS attenuates neurological function decline in rats after cerebral ischemia-reperfusion (**A**) iTBS attenuates mNSS decline in rats after cerebral ischemia-reperfusion. *n* = 6/group, ^***^P < 0.001 vs. Sham group, ^###^*P* < 0.001 vs. MCAO/R group. (**B**) iTBS attenuated cerebral ischemia-reperfusion after increased rat balance beam test scores

We assessed the degree of neurological impairment by performing mNSS and balance beam experiments on rats after MCAO/R surgery [25]. It should be noted that all post-MCAO rats had mNSS greater than 6 before iTBS was performed to be included in the experiment. The results showed that the mNSS of post-MCAO/R rats were higher than those of the sham-operated group, and iTBS treatment was able to reduce the mNSS (Fig. 4A). It indicated that iTBS reduced the severity of neurological impairment in MCAO/R rats. In addition, the results of the balance beam test showed that MCAO/R decreased the rat’s score in the balance beam experiment, representing a decrease in motor coordination and balance, which was reversed by iTBS (Fig. 4B). It is noteworthy that the rats included in the experiment all had mNSS greater than 6 after MCAO surgery. However, scores again after 7 consecutive days of iTBS stimulation showed that the MCAO/R group without iTBS stimulation had individual rats with mNSS of less than 6. This suggests that the rats have the ability to restore neurological function on their own. In conclusion, the neurological functions of rats were restored after iTBS treatment. In other words, iTBS was able to protect the neurological function after IS.

## Discussion

The current clinical treatment of IS focuses on thrombolysis within 3 to 4.5 h, and if it is not treated within this time frame, then irreversible damage to brain function can occur. This is one of the reasons why IS patients have a poorer prognosis. In addition, thrombolysis has the potential to cause ischemia-reperfusion injury. This is because of calcium overload in ischemic tissues after restoration of blood supply, as well as the high production of harmful oxygen free radicals. Finding treatment and rehabilitation for IS is always urgent and necessary. rTMS has been found to be neuroprotective and has become an important treatment nowadays. a randomized controlled trial in 2018 showed that the combined use of LF-rTMS and HF-rTMS or LF-rTMS alone was effective in promoting motor recovery of the upper limb in stroke patients. Among them, the combined use of LF-rTMS and HF-rTMS was more obvious for the improvement of motor function [26]. It is currently believed that the mechanism of action of rTMS is mainly related to the regulation of neural excitability. In addition, it also produces Long-term potentiation (LTP), which affects synaptic plasticity [27]. This process is accompanied by changes in calcium-responsive signaling pathway responses, neurotransmitter release, brain-derived neurotrophic factor production, and gene activity [28].

iTBS is a new mode of rTMS, in which the time used for burst stimulation is shorter and the induced effect is more long-lasting compared to traditional rTMS. This new treatment mode shortens the treatment time and reduces the wear and tear of the instrument to a great extent [19]. IS patients may have dysfunctions in several aspects, and the brain regions governing these functions are different, and the accurate localization of the TMS coils to the corresponding brain functional regions is the key to exerting the therapeutic effect. When treating patients in the clinic, the localization cap combined with the functional response area localization method is mostly used to determine the site, and then the figure-of-eight coil is aligned with this precise site for stimulation. In the case of iTBS stimulation in rats, the coil is placed in the center of the head because the head is too small for precise positioning. Perhaps in the future, a more precise apparatus can be designed for animal experiments to improve the limitations of the experiment. Several trials have demonstrated the effectiveness of iTBS in the treatment of stroke. First, it can improve the motor function of patients’ upper limbs [29]; second, it can also promote the recovery of gait and balance [30]; in addition, it can effectively improve swallowing function [31]. On this basis, the present study further explored the mechanism by which the iTBS mode exerts neuroprotective effects and demonstrated that it could attenuate nerve injury after ischemia-reperfusion through ERS and ferroptosis, and improve the motor coordination and balance ability of rats.

It is common in clinical practice to see patients who still have sequelae after undergoing thrombolytic therapy, including motor, sensory, speech, psychological, and cognitive functions that are impaired to varying degrees. Therefore, we used MCAO/R model rats to simulate reperfusion injury. The ipsilateral hemisphere of the rats was treated with iTBS twice a day for 7 consecutive days on the day following the MCAO surgery. The data demonstrated that iTBS was able to alleviate neurological injury in ischemia-reperfusion rats, including rescuing neuronal apoptosis and neurological deficits. mNSS proved the improvement of neurological function, whereas balance beam experiments proved the improvement of motor and balance function in rats. Meanwhile, our study further showed that the neuroprotective effects exerted were likely due to the attenuation of ERS and ferroptosis after ischemia-reperfusion injury. Three stress sensors located in the endoplasmic reticulum membrane, IRE1, PERK, and ATF6, as well as the chaperone protein GRP78, which is resident in the ER, are markers of ERS occurrence. It has been extensively demonstrated that the UPR is triggered by ERS to maintain protein homeostasis, which activates three stress sensors located in the endoplasmic reticulum membrane: IRE1, PERK, and ATF6 [8]. ERS overactivation activates both autophagy and apoptosis, the two pathways of cell death, and triggers cell death. Among them, IRE1 mediates the phosphorylation of the anti-apoptotic B-cell lymphoma-2 (Bcl-2) by initiating one of the upstream regulatory components of autophagy, the JNK signaling pathway [32]. When Bcl-2 binds to the autophagy gene Beclin-1 and is in a state of equilibrium, moderate autophagy is able to protect neuronal cells and attenuate brain damage [33]. Bcl-2 phosphorylation leads to the dissociation of Beclin1/Bcl-2 and the up-regulation of the level of Beclin-1, which activates autophagy. Excessive autophagy leads to neuronal cell death [34]. Activated PERK induces the key transcription factor ATF4 via eIF2α phosphorylation, followed by activation of CHOP (C/EBP homologous protein) to regulate ATG5 or direct regulation of ATG12 to promote autophagy [35]. In addition, activation of ATF6 can similarly promote autophagy via CHOP [36]. Overall, three stress sensors, IRE1, PERK, and ATF6, are activated during ERS to upregulate multiple autophagy-related genes. Excessive ERS not only increases autophagy but also induces apoptosis, which is associated with the activation of proteins CHOP and caspase-12 downstream of IRE1, PERK, and ATF6. Glucose-regulated protein 78 (GRP78), which is homologous to heat shock protein 70 (HSP70), is a chaperone protein resident in ER. GRP78 promotes proper protein folding and assembly and induces UPR, which is one of the markers of ERS response [37]. The reduction in the levels of these markers, IRE1, PERK, ATF6, and GRP78, after intervention with iTBS, represents an attenuation of ERS. Such results provide a theoretical basis for the mechanism by which the iTBS model attenuates reperfusion injury after ischemic stroke.

Activation of ferroptosis after cerebral ischemia is associated with increased lipid peroxidation, ultimately leading to neuronal death [13]. Inhibition of ferroptosis reverses ischemic injury [38] and has great potential in the treatment of IS. The System Xc-GSH-GPX4 axis plays an inhibitory role in ferroptosis [39], and GPX4 is able to convert and detoxify cytotoxic lipid hydroperoxides (PLOOH) [40]. In other words, GPX4 upregulation inhibits ferroptosis, while GPX4 downregulation promotes ferroptosis. a 2014 article in Cell tested and analyzed a series of genes associated with ferroptosis and showed that PTGS2, a gene encoding cyclooxygenase2 (COX2), was the gene that was upregulated the most after the onset of ferroptosis; however, inhibition of PTGS2 did not inhibit the onset of ferroptosis [41]. This suggests that COX-2 or PTGS2 can only be used as a marker for the onset of ferroptosis, but not as an effective target to inhibit ferroptosis. The increase in the level of GPX4 in the antioxidant system of iTBS-treated rats and the decrease in the level of PTGS2, a marker of ferroptosis, indicate that iTBS reduced the occurrence of ferroptosis.

A large number of cells in the ischemic penumbra undergo apoptosis after I/R injury, and the number of apoptotic neurons is closely related to the magnitude of the final damage. We assessed apoptosis in cortical tissues by measuring CHOP and Bcl-2 levels as a means of determining whether brain damage was attenuated. The Bcl-2 gene family plays a large role in the regulation of apoptosis, with those that promote apoptosis referred to as pro-apoptotic proteins, and those that inhibit apoptosis referred to as anti-apoptotic proteins [42, 43].Bcl-2 is an anti-apoptotic protein that regulates mitochondrial membrane potential (MMP) and the opening of the mitochondrial outer membrane permeable transporter pore (mPTP) pore complex through the mitochondrial pathway [44, 45], or controls Ca^2+^ in a stable range to inhibit apoptosis through the endoplasmic reticulum pathway [46]. Sustained ERS can induce apoptosis due to the upregulation of CHOP expression by three stress sensors through different pathways. Subsequently, CHOP mediates endogenous apoptosis by inhibiting the expression of the anti-apoptotic protein Bcl-2. In addition to this, CHOP also triggers caspase-8-related cascade reactions to mediate exogenous apoptosis [47]. Our study found that iTBS was able to down-regulate the level of CHOP and up-regulate the level of Bcl-2, attenuating neuronal apoptosis in rats after cerebral ischemia-reperfusion.

ERS and ferroptosis are undoubtedly involved in the development of injury after cerebral ischemia-reperfusion. Our work is basic and simple, aiming to preliminarily explore whether iTBS plays a neuroprotective role in IS and whether its mechanism is related to the inhibition of ERS and ferroptosis. It is well known that sustained activation of ERS induces apoptosis. This is because three stress sensors upregulate CHOP expression, which subsequently mediates apoptosis via endogenous or exogenous pathways. In other words, excessive ERS leads to apoptosis, and together with ferroptosis, there are already at least two cell death pathways involved in the process of ischemia-reperfusion injury. A recent study found that ferroptosis in diabetic myocardial ischemia-reperfusion injury was caused by ERS [48]. However, whether there is an interaction between ERS and ferroptosis after cerebral ischemia-reperfusion has not been demonstrated. To validate such an idea would require the addition of an inhibitor or activator of ERS and then testing the levels of ferroptosis markers. Similarly, our data can only suggest that iTBS ameliorated both ERS and ferroptosis, which in turn mitigated apoptosis as well as the decline in neurological function. Whether the beneficial effects of iTBS are due to the attenuation of ferroptosis as a result of inhibition of ERS still needs to be demonstrated subsequently, and the signaling pathways involved need to be further investigated. Another cell death pathway, pyroptosis, has been shown to be inhibited by iTBS through the TLR4/NFκB/NLRP3 signaling pathway [21]. Such results suggest that perhaps there is an interactive relationship between apoptosis, pyroptosis, autophagy, and ferroptosis. iTBS inhibition of one cell death pathway can simultaneously inhibit other cell death pathways, the mechanism of action of which is very complex and still needs to be explored in depth.

The treatment time window and the risk of complications limit the outcome of IS patients, and it has become difficult to make a breakthrough with existing treatments. Currently, immune-mediated inflammatory response has become a therapeutic target of wide interest to researchers. Recent studies have confirmed that the inflammatory response and the intrinsic immunity of the central nervous system play a key regulatory role in overall pathogenesis, and macrophages and natural killer (NK) cells are the two main types of intrinsic immune cells. Macrophages are distributed in different tissues of the human body, and microglia, as macrophages in the central nervous system (CNS), are the first and most important line of immune defense in the CNS involved in active defense and are a very central class of cell types in the CNS to maintain homeostasis and health. Microglia are cells in a dynamic process. On the one hand, in the whole life cycle of the organism, it will continue to survive and die but also be accompanied by newborn regeneration. A dynamic equilibrium is reached between the two to maintain a stable cell number. This process, called microglia turnover, is one of the central mechanisms for maintaining homeostasis. Microglia can be activated into two states, producing cytotoxic or neuroprotective effects, respectively. In the early stage of ischemic stroke, activated M2 microglia show neuroprotective effects on the brain by phagocytosis of neuronal fragments and secretion of various trophic factors, which improve the long-term neuro prognosis after stroke, but overactivation may also exacerbate neuronal death, and microglia are gradually polarized to the M1 type, which exerts neuroinjurious effects mainly through secretion of some proinflammatory factors. M2-type microglia can participate in post-stroke neuroprotective mechanisms and their derived exosomes protect the brain from ischemia-reperfusion injury [49]. In addition, extracellular vesicles derived from M2-type microglia can promote white matter repair after ischemic stroke in mice [50], which is key to the recovery of cognitive and neurological functions after ischemic stroke. Modulating the activation state of macrophages using clinical drugs, gene modulation, or cell transplantation in an appropriate time window can improve the inflammatory environment and convert M1-type microglia to M2-type, which is an effective approach to treat the disease. iTBS has been shown to modulate the polarization of microglia and improve the motor function of mice with cerebral ischemia [21]. Natural killer (NK) cells are innate lymphocytes that can be rapidly mobilized in the early stages of the immune response, and NK cells in the bloodstream are rapidly summoned into the brain tissue, especially at the site of injury, within 30 min after stroke. nKG2A is an inhibitory receptor expressed on the surface of NK cells, and nKG2D is an activating receptor, in which there is no significant ipsilateral and contralateral expression of nKG2A The difference suggests that left basal ganglia infarction caused not only ipsilateral but also contralateral immune responses, thus showing that stroke is not a localized injury but an active whole-brain immunity. As a result, some patients have depression and cognitive changes secondary to an injury that is not severe and does not damage a vital part of the brain. This explains why it is necessary to carry out simultaneous bilateral rehabilitation after stroke, and when applying TMS to post-stroke patients, in addition to stimulating the affected cerebral cortex, stimulating the healthy cerebral cortex might likewise improve functional recovery. Our present work focused on ferroptosis and apoptosis induced by ERS and did not examine immune cells as well as inflammatory factors. It is therefore not possible to speculate whether iTBS reduces cell death by having an effect on the immune system. The interaction between the immune system and the nervous system is also a target for us to carry out the next research.

## Data Availability

The relevant data are included in the article.
